# The effects of mood disorders and childhood trauma on fear of positive and negative evaluation

**DOI:** 10.1016/j.actpsy.2022.103603

**Published:** 2022-05-04

**Authors:** Mora M. Lucero, Skye Satz, Rachel Miceli, Holly A. Swartz, Anna Manelis

**Affiliations:** Department of Psychiatry, Western Psychiatric Institute and Clinic, University of Pittsburgh Medical Center, University of Pittsburgh, Pittsburgh, PA, USA

**Keywords:** Bipolar disorder, Depressive disorders, Childhood trauma, Fear of positive evaluation, Fear of negative evaluation trauma

## Abstract

Fear of positive and negative evaluation is maladaptive and may result in psychosocial dysfunction. Although being diagnosed with mood disorders or experiencing childhood trauma may potentially affect fear of evaluation, previous studies examined this phenomenon mostly in social anxiety disorders. To fill this gap, we investigated the relationship between childhood trauma and fear of positive and negative evaluation in individuals with bipolar disorder (BD), depressive disorders (DD), and healthy controls (HC). 43 individuals with BD, 89 with DD, and 65 HC completed clinical interviews and self-report assessments. The relationship between participants’ diagnoses and presence of trauma on fear of positive and negative evaluation was examined using ANCOVA. Independently of experiencing childhood trauma, fear of positive evaluation was significantly higher in individuals with mood disorders vs. HC. Fear of negative evaluation was significantly associated with diagnosis-by-trauma interaction. Significantly lower scores were observed in individuals with BD without childhood trauma compared to those with childhood trauma and individuals with DD. Compared to HC, more individuals with mood disorders experienced childhood trauma. While experiencing childhood trauma may increase vulnerability to mood disorders in general, it is especially detrimental for individuals with BD by increasing the risk for developing a fear of negative evaluation.

## 1. Introduction

Depressive disorders (DD; e.g., major depressive and persistent depressive disorders) and bipolar disorder (BD) are universally recognized as debilitating illnesses leading to poor emotional, social, and cognitive functioning and overall impairments in quality of life. As consequences to severe symptomology (Judd et al., 2008; Shippee et al., 2011), individuals with mood disorders exhibit social skill deficits (Kupferberg et al., 2016), more strained interpersonal relationships (Elliott & Briere, 1995), exhausted empathic reactions (Oakley et al., 2012; Smith & Rose, 2011), low self-esteem (Shahar & Davidson, 2003), and aberrant future-directed thinking (Mathews & MacLeod, 2005) compared to healthy controls (HC).

Social and emotional dysfunction in individuals with mood disorders (Orhan et al., 2018; Rhebergen et al., 2010) can lead to increased social rejection sensitivity (Ehnvall et al., 2014) and social anhedonia (Akiskal et al., 2006; Kupferberg et al., 2016), which, in turn, could result in the fear of evaluation and an inability to effectively use feedback from other people. Individuals with elevated fear of negative evaluation tend to be more anxious about how others view them, leading to an avoidance of socially evaluative situations that could result in being rejected (Carter et al., 2012; Watson & Friend, 1969). The detrimental consequence of this fear is a development of cognitive bias that others will recognize imperfections in an individual’s appearance or behavior. Such bias may stimulate heightened social anxiety leading to social avoidance of perceived negative interactions (Carter et al., 2012; Rapee & Heimberg, 1997; Weeks et al., 2010). Individuals with elevated fear of positive evaluation tend to feel more apprehensively self-conscious and socially incomparable to others (Weeks et al., 2008). These individuals may develop a cognitive bias that if they perform well, they must exceed previous achievements to satisfy the standards set by others (Carter et al., 2012). Therefore, fear of positive evaluation may negatively impact one’s belief in their ability to match these standards and expectations.

Despite extensive evidence for significant psychosocial dysfunction and biased social cognition in mood disorders (Caouette & Guyer, 2016; Schulz et al., 2008), fears of positive and negative evaluation were not examined in the context of these disorders. Instead, previous studies mostly focused on understanding the fear of positive evaluation (Fergus et al., 2009; Fredrick & Luebbe, 2020), and negative evaluation (Rapee & Heimberg, 1997; Rodebaugh et al., 2004) in the context of social anxiety disorders (SAD). Considering that fear of evaluation may be related to other mental illnesses (Downey & Feldman, 1996) and that fear of social rejection is a risk factor for developing depression (Kumar et al., 2017; Slavich et al., 2010), it is critically important to examine the relationship between mood disorders and the fear of evaluation. Understanding this relationship may inform the development of effective preventative and treatment strategies to help affected individuals remain active and productive members of society.

Epidemiological research and neurobiological research have proposed that the onset of DD and BD often occurs during childhood and adolescence (Birmaher et al., 2014; Fleisher & Katz, 2001). One major risk factor for the development of early depressive symptomology is exposure to childhood trauma (Aas et al., 2016). Several meta-analyses (Mandelli et al., 2015; Matheson et al., 2013), cross-sectional (Molnar et al., 2001; Sugaya et al., 2012) and longitudinal studies (Tanskanen et al., 2004) have demonstrated that people with histories of childhood trauma are roughly three times more likely to have psychiatric illnesses during adulthood than people without trauma histories.

While there is clear evidence that childhood trauma affects depressive symptoms and a concept of self (Reyes, 2008; Turner et al., 2010), no previous study has investigated the relationships among childhood trauma, mood disorder diagnosis, and fear of evaluation. The goal of this study was to bridge this gap by investigating the effect of childhood trauma on fear of positive and negative evaluation in individuals with BD, DD, and HC. We hypothesized that, compared to HC, individuals with mood disorders would report greater occurrences of traumatic events and greater fear of positive and negative evaluation. We also hypothesized that fear of evaluation would be greater in individuals with childhood trauma experiences and that this relationship would differ between BD and DD. In accordance with previous literature, we also explored the relationships between fear of evaluation and trait anxiety indicating people’s tendency to perceive situations as threatening (Spielberger, 1983). We hypothesized that higher fears of being evaluated would be related to higher trait anxiety across diagnosis.

## 2. Methods

### 2.1. Participants

The study was approved by the Institutional Review Board. Written informed consent was obtained from all participants. 197 participants between 18 and 45 years of age were recruited from community, including universities, and counseling and medical centers. Healthy controls (*n* = 65) had no personal or familial history of psychiatric disorders. Symptomatic individuals met DSM 5 criteria for depressive disorders (DD, i.e., major depression or persistent depressive disorders; *n* = 89) and bipolar disorder type I and type II (*n* = 43).

### 2.2. Assessments

#### 2.2.1. Clinical assessments

All mood disorders and comorbid disorders diagnoses were made in accordance with DSM-5 criteria using Structured Clinical Interview (First et al., 2015) by a trained clinician and confirmed by a study psychiatrist. Current depression symptoms were assessed using the Hamilton Depression Rating Scale (HDRS-25; Hamilton, 1960) and current mania symptoms were assessed using the Young Mania Rating Scale (YMRS; Young et al., 1978). The State-Trait Anxiety Inventory Form Y (STAIY) (Spielberger, 1983) was used to assess trait anxiety. On all scales, higher scores indicated more severe symptomatology.

#### 2.2.2. Trauma assessment

Childhood trauma was evaluated using the Childhood Traumatic Events Scale (Pennebaker & Susman, 1988). Participants indicated whether they experienced such traumatic events as the death of a loved one, a major upheaval between parents, a traumatic sexual experience, being a victim of violence, or having been extremely ill or injured prior to the age of 17. For each experienced traumatic event, a participant indicated how traumatic the event was on a scale from 1 (not at all traumatic) to 7 (extremely traumatic). Given that traumatic intensity is difficult to calculate when a person experienced several instances of a particular traumatic event, when the event lasted for months or years, or when the trauma occurred during early childhood, we generated a categorical ‘yes’/’no’ score for the presence of trauma (yes/no trauma) without considering its type and intensity.

#### 2.2.3. Fear of evaluation assessments

Fear of positive evaluation was assessed using the Fear of Positive Evaluation self-report questionnaire (FPE; Weeks et al., 2008), which measures apprehension about being favorably evaluated by others (i.e., *I feel uneasy when I receive praise from authority figures*) on a 10-point Likert Scale, from 0 (not true at all) to 9 (very true). A total FPE score was computed as a sum of all responses without items 5 and 10 based on previous recommendations (Weeks et al., 2008) and could range from 0 to 72.

Fear of negative evaluation was assessed using the 12-item Brief Fear of Negative Evaluation self-report questionnaire (BFNE; Leary, 1983), which measures anxiety and distress about being negatively evaluated by others (i.e., *I am afraid that people will find fault in me*) on a 5-point Likert Scale, from 1 (not at all) to 5 (extremely). A total BFNE score was computed as a sum of all responses and could range from 10 and 60.

### 2.3. Data analyses

#### 2.3.1. Main model analyses

All demographic and clinical characteristics were compared among BD, DD, and HC groups using Analysis of Variance (ANOVA) tests in R (https://www.r-project.org). A 3 (BD/DD/HC)-by-2 (Trauma presence/absence) ANCOVA with age, sex, and IQ as covariates was used to evaluate the effect of mood disorders and the presence of childhood trauma on the fear of positive evaluation and separately on the fear of negative evaluation. The estimated contrasts and means were calculated using the *modelbased* package (Makowski et al., 2020) in R. When appropriate, the *p*-values were corrected for multiple comparisons using Benjamin and Hochberg’s False Discovery Rate (FDR; Benjamini, 2010).

#### 2.3.2. Exploratory analyses

Given previous findings of the relationship between anxiety and fear of negative evaluation (Rapee & Heimberg, 1997; Watson & Friend, 1969) and fear of positive evaluation (Fredrick & Luebbe, 2020; Weeks et al., 2008, 2010), we evaluated the relationship between trait anxiety (STAIY2 scores) and fears of positive and negative evaluation. In one set of models, trait anxiety was used as an additional covariate, while in the other set of models, trait anxiety was used as a third interaction term in the model described in Section 2.3.1.

In addition, we examined how current symptoms of depression (the HRSD-25 scores) were related to the fear of positive and negative evaluation in individuals with BD and DD. The analysis conducted across these two groups used the HRSD-25 scores as a third interaction term in the model described in Section 2.3.1.

## 3. Results

### 3.1. Demographic and clinical characteristic

The three groups of participants did not differ in gender composition, but significantly differed in age and IQ (Table 1). BD were younger than HC (FDR-corrected *p* = 0.03) but did not differ from DD who did not differ from HC. Participants with DD and BD had significantly higher HRSD-25, YMRS, and STAIY2 scores than HC (all FDR-corrected *p*-values<0.001) but did not differ from each other (Table 1). The proportion of participants who experienced childhood trauma differed among the three groups mostly because a smaller percent of HC experienced trauma prior to the age of 17 (52%), compared to those with BD (70%) and DD (83%).

### 3.2. Effect of trauma on fear of evaluation

#### 3.2.1. Fear of positive evaluation

**Fig. 1.**
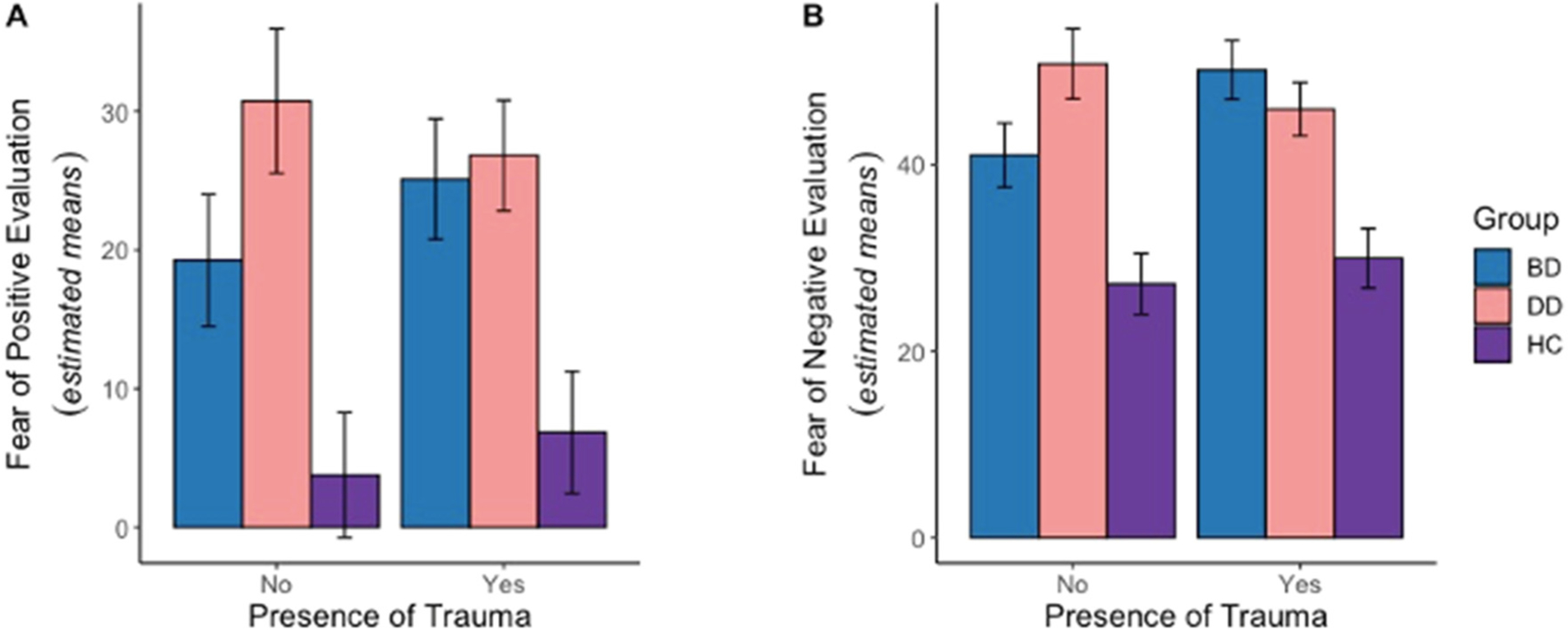
Estimated means of fear of positive evaluation scores (A) and fear of negative evaluation scores (B) in BD, DD, and HC with and without childhood trauma.

A 2-way ANCOVA revealed a significant main effect of group [*F*(2,187) = 46.7, *p* < 0.001], but no main effect of trauma or group-by-trauma interaction (Fig. 1A). Individuals with BD and DD had higher FPE scores compared to HC independently of childhood trauma experiences (FDR-corrected-*p*-values<0.05, Table S1).

#### 3.2.2. Brief fear of negative evaluation

A 2-way ANCOVA revealed a significant group-by-trauma interaction effect [*F*(2,187) = 4.95, *p* < 0.01] and a main effect of group [*F*(2,187) = 82.4, *p* < 0.001] (Fig. 1B). Individuals with BD who did not experience childhood trauma had significantly lower BFNE scores than those with childhood trauma experiences (FDR-corrected-*p*-values = 0.014) and individuals with DD without childhood trauma (FDR-corrected-*p* = 0.022). Individuals with BD and DD had higher BFNE scores compared to HC (FDR-corrected-*p*-values<0.05, Table S2).

### 3.3. Results of the exploratory analyses

#### 3.3.1. The effect of trait anxiety on fear of evaluation

**Fig. 2.**
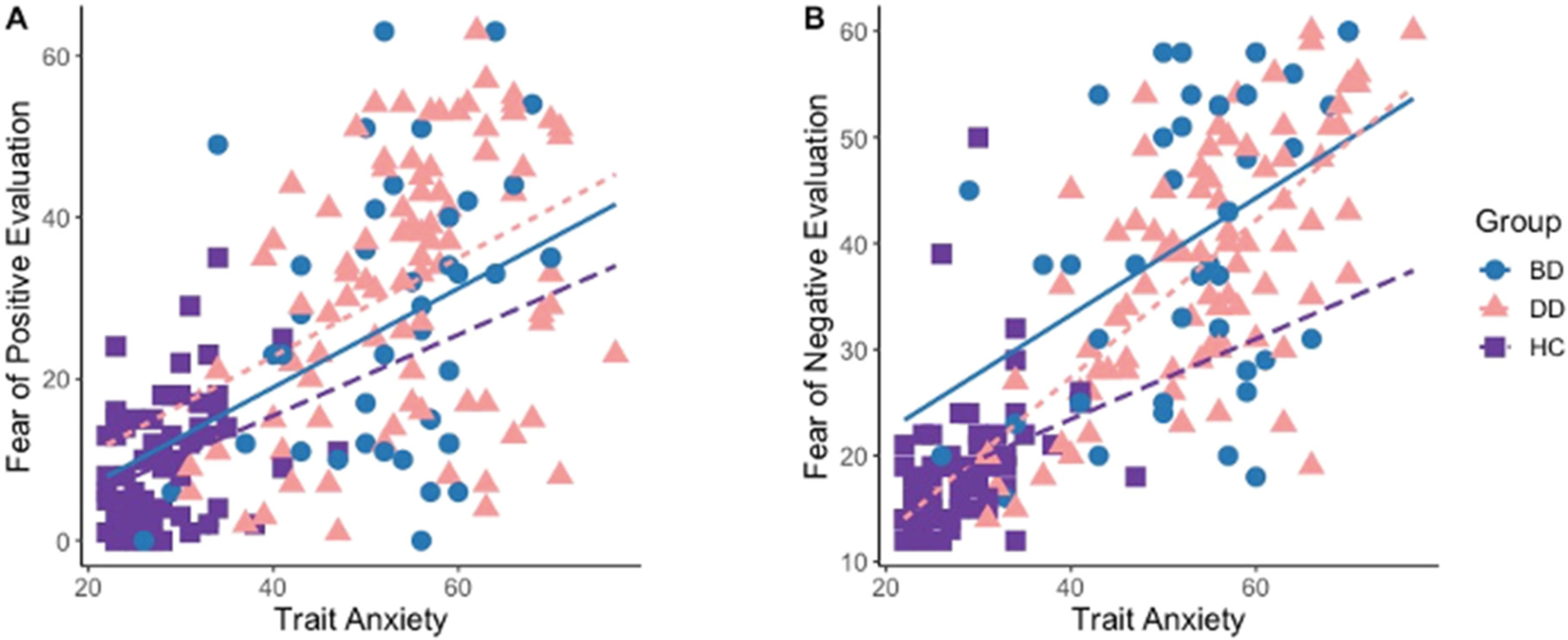
Main effect of trait anxiety scores on a fear of positive evaluation (A), and fear of negative evaluation (B) in BD, DD, and HC.

Entering the trait anxiety either as a covariate or as an interaction term (group-by-trauma-by-anxiety) into the models described above, did not change the main findings: the main effect of group on the FPE scores [covariate: *F*(2,185) = 54.7, *p* < 0.001; interaction: *F*(2,180) = 53.96, *p* < 0.001] and the group-by-trauma interaction [covariate: *F*(2,185) = 6.7, *p* < 0.01; interaction: *F*(2,180) = 6.7, *p* < 0.01] and the main effect of group [*F*(2,185) = 116.3, *p* < 0.001; interaction: *F*(2,180) = 115.5, *p* < 0.001] on the FNE scores remained significant. While there was a significant effect of the trait anxiety scores on both FPE [covariate: *F*(1,185) = 33, *p* < 0.001; interaction: *F*(1,180) = 32.6, *p* < 0.001] and FNE [covariate: *F*(1,185) = 90.7, *p* < 0.001; interaction: *F*(1,180) = 90.1, *p* < 0.001], no significant group-by-anxiety, trauma-by-anxiety, or group-by-trauma-by-anxiety interactions were observed (Fig. 2).

#### 3.3.2. The effect of current depression symptoms on fear of evaluation in the BD and DD groups

A 3-way ANCOVA did not reveal significant effects of the HRSD-25 scores [*F*(1,120) = 3.2, *p* = 0.08], HRSD-25-by-group [*F*(1,120) = 0.02, *p* = 0.9], HRSD-25-by-trauma [*F*(1,120) = 2.5, *p* = 0.1], or HRSD-25-by-group-by-trauma [*F*(1,120) = 0.1, *p* = 0.8] interactions on FPE. A 3-way ANCOVA revealed a significant HRSD-25-by-group interaction effect on the FNE scores [*F*(1,120) = 5.3, *p* = 0.02] that was driven by a significant positive slope in the UD [*t*(122) = 2.7, *p* = 0.009] but not BD [*t*(122) = −0.42.7, *p* = 0.7] group. There were no significant effects of the HRSD-25 scores [*F*(1,120) = 3.3, *p* = 0.07], HRSD-25-by-trauma [*F*(1,120) = 0.14, p = 0.7], or HRSD-25-by-group-by-trauma [*F*(1,120) = 1.4, *p* = 0.24] interactions on FNE.

## 4. Discussion

In this study, we investigated the relationships among fear of positive and negative evaluation, mood disorder diagnoses, and presence of childhood trauma. We found that both fear of positive and fear of negative evaluation were significantly higher in individuals with mood disorders, compared to healthy controls. Experiencing childhood trauma was not related to fear of positive evaluation. However, fear of negative evaluation was associated with mood disorder diagnosis when childhood trauma was also present. Specifically, those individuals with BD who did not experience childhood trauma had significantly lower fear of negative evaluation than those with trauma experiences, and those with DD without childhood trauma. Our work extends previous research on the relationship between fear of evaluation and social anxiety disorders (Fergus et al., 2009; Fredrick & Luebbe, 2020; Rapee & Heimberg, 1997; Watson & Friend, 1969) by showing that higher fears of being evaluated are related to DD and BD diagnoses. Our research also contributes to understanding the differences between BD and DD.

Consistent with previous findings and the Diathesis-Stress model, we found that higher percent of individuals with mood disorders, compared to HC, have experienced childhood trauma (Belsky & Pluess, 2009; Colodro-Conde et al., 2018). The Diathesis-Stress model proposes that depressive psychopathology is engendered by both genetic predisposition and environmental stress. Experiencing trauma prior to the age of 17 is a major environmental stressor that increases risk of developing mental illnesses (Mauritz et al., 2013; Molnar et al., 2001; Sugaya et al., 2012). Independent of childhood trauma history, both individuals with BD and those with DD reported greater fears of positive evaluation compared to healthy individuals. A scar model of depression suggests that experiencing mental illness results in personality changes and further decrease in self-esteem (Rohde et al., 1990; Shahar & Davidson, 2003). Specifically, having more severe depressive symptoms predicted a self-esteem decrease in the future (Shahar & Davidson, 2003). In the context of our findings, greater fear of positive evaluation can be interpreted as a ‘scar’ from experiencing depression independently of having or not having hypo/mania episodes. For example, affected individuals may challenge praise given from others thinking that they are undeserving of recognition or that others would think them underserving.

Fear of negative evaluation depended on both a specific mood disorder diagnosis and the presence of childhood trauma experiences. Bipolar disorder is characterized by episodes of hypo/mania during which affected individuals may have inflated self-esteem, be engage in risky behaviors, and become insensitive to criticism by others (Masland et al., 2015; Scott et al., 2012). In contrast, experiencing depressive episodes may increase apprehension of being negatively evaluated others and impede people’s ability to accept and respond to criticism. It is possible that in the absence of childhood trauma, experiencing high self-esteem during hypo/mania episodes may help to reduce fear of negative evaluation in individuals with BD in general. However, experiencing childhood trauma may trigger negative cognitions about oneself and the world, thus influencing how individuals with BD interpret their experiences (Cromer & Smyth, 2010; Frissa et al., 2016). Our findings are consistent with this account and suggest that having childhood trauma is especially detrimental for individuals with BD by increasing fear of being negatively evaluated by others. The results of the exploratory analysis extend previous findings by showing that fear of negative evaluation is positively associated with current depression severity in DD but not BD.

Most previous research on fear of negative (Rapee & Heimberg, 1997; Watson & Friend, 1969) and positive (Fredrick & Luebbe, 2020; Weeks et al., 2008, 2010) evaluation examined these phenomena in the context of social anxiety disorders. These studies suggested that socially anxious people believed that they would be evaluated negatively in social situations (Schulz et al., 2008) and that social anxiety mediated the fear of positive evaluation (Weeks et al., 2008). While we were not able to compare mood disordered individuals with and without comorbid social anxiety disorders and with and without childhood trauma due to small sample sizes, we explored the effect of trait anxiety on fears of positive and negative evaluation. These analyses revealed a significant main effect of trait anxiety on fears of positive and negative evaluation, but no significant interaction effect with diagnostic group or childhood trauma. Adding trait anxiety to the model did not change the significant group and group-by-trauma interaction effects described above. These findings suggest that mood disorder diagnosis is the main contributing factor associated with fears of positive and negative evaluation and that trait anxiety is likely not the driving force in this relationship. Future research should identify factors contributing to elevated fears of positive and negative evaluation in the context of mood disorders and explore ways to lessen those fears.

One limitation of this study was a relatively small sample size of the BD group. We partially addressed this concern by reporting the results that remained significant after correcting for multiple comparison. The other limitation of this study—and other studies on childhood trauma—was that childhood memories could be affected by recall bias, a person’s subjective perspective on self-relevant events (McNally, 2003) that can be affectively congruent with current emotional state (Bower, 1981). Such recall bias may influence accuracy and willingness to disclose a traumatic event, thus resulting in underreporting (or inaccurate reporting) of traumatic experiences. Severity of a traumatic event was difficult to objectively calculate due to frequency and duration. One traumatic event (e.g., rape) versus multiple instances of the same traumatic category (e.g., sexual assault, sexual harassment, exposure to indecent pictures), as well as long-lasting, repetitive traumatic events (e.g., child abuse that lasts for years) could all be rated as equally intense. However, the effects of these events on personality and mental illness development could be drastically different. Therefore, another limitation of this study was the dichotomous approach to childhood trauma experiences that did not account for the perceived intensity of the traumatic events.

Part of the challenge with assessing trauma is that there is no agreed-upon strategy for defining the variable and cumulative experiences of trauma. Trauma is known to be physically or emotionally distressing with lasting adverse effects. But the concept of *distress* itself is abstract and not necessarily associated with psychological dysfunction. Distinguishing between normal/healthy versus abnormal/pathological reactions to a traumatic event is also subject to various interpretations. For example, exposure to parental divorce may give rise to emotional distress, but this distress may not manifest itself as mental illness (McNally, 2003), nor be identified as a severe traumatic experience. In some cases, it may be associated with resilience (Cheng et al., 2014). Future research should investigate objective ways to distinguish adaptive from maladaptive responses.

In conclusion, this study revealed that fears of positive and negative evaluation are higher in individuals with mood disorders. A higher percentage of individuals with BD and DD, compared to their healthy counterparts, reported childhood trauma that occurred prior to the age of 17, which is consistent with the Diathesis-Stress model. Our results suggest that experiencing childhood trauma maybe especially detrimental for individuals with BD by increasing the risk for developing a fear of negative evaluation in these individuals. Considering that both fear or positive and fear of negative evaluation are maladaptive and may result in social and professional dysfunction, progress in understanding how specific mood disorders and childhood trauma contribute to development of these phenomena is critical for building effective psychosocial treatment approaches and mitigation of developing psycho-pathological symptoms in adulthood.

## Supplementary Material

1

## Figures and Tables

**Table 1:** Demographic and clinical characteristics.

	BD	DD	HC	Statistics
N	43	89	65	
Number of female/male/other	32/8/3	66/22/1	50/15/0	chi2(4) = 7.38, *p* = 0.12
N with trauma prior to the age of 17	30	74	34	chi2(2) = 17.03, *p <* 0.001
Age	25.53 (0.72)	27.39 (0.7)	28.71 (0.77)	*F*(2,194) = 3.46, *p* = 0.03
IQ (NART)	109.41 (1.02)	108.62 (0.8)	106.22 (0.83)	*F*(2,194) = 3.25, *p* = 0.04
HRSD-25	11.58 (1.26)	13.61 (0.79)	1.69 (0.24)	*F*(2,194) = 68.46, *p <* 0.001
YMRS	2 (0.47)	1.47 (0.24)	0.25 (0.09)	*F*(2,194) = 10.53, *p <* 0.001
STAIY2	52.51 (1.6)	54.19 (1.12)	28.23 (0.63)	*F*(2,193) = 170.32, *p <* 0.001
N with comorbid anxiety disorders (no trauma/trauma)	4/11	8/35	na	
N without comorbid anxiety disorders (no trauma/trauma)	9/19	7/39		

Note. The table reports the mean and standard error of mean (SE) in parenthesis.
