# Supplementary material for: The effects of mood disorders and childhood trauma on fear of positive and negative evaluation
Source: Acta Psychol (Amst). Author manuscript; Available in PMC 2022 Jul 1. (PMC9189689; doi:10.1016/j.actpsy.2022.103603)
Supplement: 1 [file NIHMS1812595-supplement-1.docx]

**Title**: The effects of mood disorders and childhood trauma on fear of positive and negative evaluation

**SUPPLEMENTAL MATERIALS**

**Table S1.** Estimated Contrasts for Fear of Positive Evaluation

|  |  | **Difference** | **SE** | **95% Confidence Interval** | **t(187)** | **p-value** |
| --- | --- | --- | --- | --- | --- | --- |
| BD without trauma | BD yes trauma | -5.85 | 4.76 | [-20.01, 8.31] | -1.23 | 0.276 |
|  | HC without trauma | 15.49 | 4.87 | [ 1.01, 29.96] | 3.18 | 0.004 |
|  | HC yes trauma | 12.41 | 4.83 | [ -1.94, 26.76] | 2.57 | 0.02 |
|  | DD without trauma | -11.48 | 5.50 | [-27.85, 4.88] | -2.09 | 0.064 |
|  | DD yes trauma | -7.55 | 4.38 | [-20.56, 5.46] | -1.73 | 0.129 |
| BD yes trauma | HC yes trauma | 18.26 | 3.61 | [ 7.53, 29.00] | 5.06 | <0.001 |
|  | DD yes trauma | -1.70 | 3.04 | [-10.75, 7.35] | -0.56 | 0.577 |
| HC without trauma | BD yes trauma | -21.34 | 3.64 | [-32.16, -10.52] | -5.87 | <0.001 |
|  | HC yes trauma | -3.07 | 3.55 | [-13.63, 7.48] | -0.87 | 0.415 |
|  | DD without trauma | -26.97 | 4.42 | [-40.13, -13.82] | -6.10 | <0.001 |
|  | DD yes trauma | -23.04 | 3.04 | [-32.09, -13.99] | -7.57 | <0.001 |
| HC yes trauma | DD yes trauma | -19.97 | 2.98 | [-28.81, -11.12] | -6.71 | <0.001 |
| DD without trauma | BD yes trauma | 5.63 | 4.46 | [ -7.64, 18.91] | 1.26 | 0.276 |
|  | HC yes trauma | 23.90 | 4.38 | [ 10.86, 36.94] | 5.45 | <0.001 |
|  | DD yes trauma | 3.93 | 3.99 | [ -7.92, 15.78] | 0.99 | 0.375 |

**Table S2.** Estimated Contrasts for Fear of Negative Evaluation

|  |  | **Difference** | **SE** | **95% Confidence Interval** | **t(187)** | **p-value** |
| --- | --- | --- | --- | --- | --- | --- |
| BD without trauma | BD yes trauma | -9.15 | 3.45 | [-19.39, 1.09] | -2.66 | 0.014 |
|  | HC without trauma | 13.81 | 3.52 | [ 3.34, 24.28] | 3.92 | <0.001 |
|  | HC yes trauma | 11.06 | 3.49 | [ 0.67, 21.44] | 3.17 | 0.003 |
|  | DD without trauma | -9.80 | 3.98 | [-21.63, 2.04] | -2.46 | 0.022 |
|  | DD yes trauma | -4.94 | 3.16 | [-14.35, 4.47] | -1.56 | 0.139 |
| BD yes trauma | HC yes trauma | 20.20 | 2.61 | [ 12.44, 27.97] | 7.74 | <0.001 |
|  | DD yes trauma | 4.21 | 2.20 | [ -2.34, 10.76] | 1.91 | 0.078 |
| HC without trauma | BD yes trauma | -22.96 | 2.63 | [-30.79, -15.14] | -8.73 | <0.001 |
|  | HC yes trauma | -2.76 | 2.57 | [-10.39, 4.87] | -1.07 | 0.304 |
|  | DD without trauma | -23.61 | 3.20 | [-33.13, -14.09] | -7.38 | <0.001 |
|  | DD yes trauma | -18.76 | 2.20 | [-25.30, -12.21] | -8.52 | <0.001 |
| HC yes trauma | DD yes trauma | -16.00 | 2.15 | [-22.40, -9.60] | -7.43 | <0.001 |
| DD without trauma | BD yes trauma | 0.65 | 3.23 | [ -8.95, 10.25] | 0.20 | 0.841 |
|  | HC yes trauma | 20.85 | 3.17 | [ 11.42, 30.28] | 6.57 | <0.001 |
|  | DD yes trauma | 4.86 | 2.88 | [ -3.72, 13.43] | 1.68 | 0117 |
